# Changes in Non-Enzymatic Antioxidants in the Blood Following Anaerobic Exercise in Men and Women

**DOI:** 10.1371/journal.pone.0143499

**Published:** 2015-11-24

**Authors:** Magdalena Wiecek, Marcin Maciejczyk, Jadwiga Szymura, Zbigniew Szygula, Malgorzata Kantorowicz

**Affiliations:** 1 Department of Physiology and Biochemistry, Faculty of Physical Education and Sport, University of Physical Education in Krakow, Krakow, Poland; 2 Department of Clinical Rehabilitation, Faculty of Motor Rehabilitation, University of Physical Education in Krakow, Krakow, Poland; 3 Department of Sports Medicine and Human Nutrition, Faculty of Physical Education and Sport, University of Physical Education in Krakow, Krakow, Poland; 4 Faculty of Physical Education and Sport, University of Physical Education in Krakow, Krakow, Poland; Universidad Pablo de Olavide, Centro Andaluz de Biología del Desarrollo-CSIC, SPAIN

## Abstract

**Purpose:**

The aim of this study was to compare changes in total oxidative status (TOS), total antioxidative capacity (TAC) and the concentration of VitA, VitE, VitC, uric acid (UA), reduced (GSH) and oxidized glutathione (GSSG) in blood within 24 hours following anaerobic exercise (AnEx) among men and women.

**Methods:**

10 women and 10 men performed a 20-second bicycle sprint (AnEx). Concentrations of oxidative stress indicators were measured before AnEx and 3, 15 and 30 minutes and 1 hour afterwards. UA, GSH and GSSH were also measured 24 hours after AnEx. Lactate and H^+^ concentrations were measured before and 3 minutes after AnEx.

**Results:**

The increase in lactate and H^+^ concentrations following AnEx was similar in both sexes. Changes in the concentrations of all oxidative stress indicators were significant and did not differ between men and women. In both sexes, TOS, TAC, TOS/TAC and VitA and VitE concentrations were the highest 3 minutes, VitC concentration was the highest 30 minutes, and UA concentration was the highest 1 hour after AnEx. GSH concentration was significantly lower than the initial concentration from 15 minutes to 24 hour after AnEx. GSSG concentration was significantly higher, while the GSH/GSSG ratio was significantly lower than the initial values 1 hour and 24 hour after AnEx.

**Conclusions:**

With similar changes in lactate and H^+^ concentrations, AnEx induces the same changes in TAC, TOS, TOS/TAC and non-enzymatic antioxidants of low molecular weight in men and women. Oxidative stress lasted at least 24 hours after AnEx.

## Introduction

Peak anaerobic power is significantly higher in men than in women [1] due to a greater mass of skeletal muscles in the former [2] and differences related to the histology and metabolic activity of myocytes among the sexes [3–6]. While the share of slow-twitch and fast-twitch muscle fibers in skeletal muscles is similar in both sexes [7], men show a greater cross-sectional area of fast-twitch fibers [3], higher ratios between the cross sections of the following pairs of fiber types: IIA/I, IIB/I and IIB/IIA [8] and, consequently, a greater share of Type II (MyHC II) myosin heavy chains than women [6]. The share of Type II fibers and peak anaerobic power correlate positively with the level of post-exercise oxidative stress [9]. Oxidative stress results from a disrupted balance between oxidation processes involving reactive oxygen species (ROS) and reactive nitrogen species (RNS), and the enzymatic and non-enzymatic antioxidant defense [10]. Upregulation of ATP resynthesis during sprinting leads to a considerable increase in AMP/ATP and lactate/pyruvic acid concentration ratios, a decrease in phosphocreatine concentration and a decrease in the NAD^+^/NADH concentration ratio in myocytes [11]. In turn, the increase in AMP/ATP concentration ratio leads to an increase (following anaerobic exercise) in purine metabolism involving xanthine oxidase and a shift in the prooxidant-antioxidant balance towards oxidation, caused by excessive production of the following types of ROS: the superoxide anion radical (O_2_^•-^), hydrogen peroxide (H_2_O_2_) and the hydroxyl radical (^•^OH) [12]. ROS and RNS are also produced during myocyte contraction due to the activation of NADPH oxidase and nitric oxide synthase [12]. A significant positive correlation was observed between the increase in lactate concentration in blood plasma following exercise and the increase in total oxidative status of blood plasma expressed through lipid peroxide concentration [13]. The activity of anaerobic metabolism enzymes is greater in men than in women [4,5,8]. As a result, men display greater disruptions to the acid-base balance in the blood following anaerobic exercise [14,15]. Catecholamine concentration in the blood is also higher in men than in women following anaerobic exercise [15], which may intensify oxidative stress in the former [16]. On the other hand, a significant, yet similar in both sexes, increase in AMP-activated protein kinase (AMPK) phosphorylation was found in muscles following anaerobic exercise [17]. Because AMPK activates nitric oxide synthesis [18], its increased phosphorylation may indicate that both sexes undergo a similar post-exercise increase in RNS concentration.

Anaerobic exercise is followed by an increase in lipid peroxide concentration and the total antioxidative capacity of blood plasma, which indicates disruptions to the prooxidant antioxidant balance [19]. Even though research has observed the activation of the κB nuclear factor in the nuclei of peripheral blood mononuclear cells [20], enzymatic antioxidant defense has not yet been unambiguously shown to have a significant role in preventing the development of oxidative stress following anaerobic exercise. A study by Berzosa et al. [21] found an increase in the activity of catalase, superoxide dismutase and glutathione peroxidase following anaerobic exercise. However, a different study observed a decrease in superoxide dismutase activity and no changes in glutathione peroxidase activity [22]. It seems that in the case of anaerobic exercise, antioxidant defense results from the activity of low molecular weight non-enzymatic antioxidants, e.g., reduced glutathione, uric acid, β-carotene, α-tocopherol and ascorbic acid, which constitute a significant portion of the blood plasma’s total capacity for antioxidants [23–25].

Few studies investigated changes in human prooxidant-antioxidant balance following anaerobic exercise [26]. Most of these studies either involved only men [11,19–21,27–29] or did not take sex into consideration during data analysis [30,31].

Taking into account the production process of ROS and RNS as well as differences related to the histological structure and metabolic activity of myocytes among the sexes, the authors of this study proposed the hypothesis: post-exercise disruptions to the prooxidant-antioxidant balance in the blood are more severe in men than in women. To verify these hypotheses, oxidative stress caused by a 20-second-long bicycle sprint between men and women was compared based on changes in the total oxidative status and total antioxidative capacity of the blood and on changes in the concentration of low molecular weight non-enzymatic antioxidant 24 hours after the exercise.

## Materials and Methods

The study project was approved by the Commission for Bioethics at the Regional Medical Chamber in Krakow, Poland (opinion No. 81/KBL/OIL/2013). The study was conducted according to the Declaration of Helsinki. Study participants were informed about the aim of the study, laboratory conditions, equipment and research procedures, and gave written consent for voluntary participation in the project. The participants underwent medical qualification that involved taking their medical history, blood count and ECG screening to eliminate medical contraindications to performing maximal and anaerobic exercise. In addition, women underwent a gynecological interview to confirm that their menstrual cycles were regular and that they took no hormonal drugs. All exercise tests were conducted under the supervision of a physician specializing in sports medicine.

### Participants

Study participants comprised 10 women and 10 men aged 22.0±0.5 years and 21.6±0.4 years, respectively. All of them were healthy non-smokers, no vegetarian and were physically active (11.1±3.9 hr/week: 7.4±2.9 hr/week, 2.2±1.1 hr/week, 1.5±1.3 hr/week respectively moderate, hard, and very hard intensity), but did not engage in any sports discipline. The participants’physical activity was assessed using a Seven Days Physical Activity Recall (7-day PAR) questionnaire [32].

The participants’ body mass (BM), body height (BH) and relative values of maximal oxygen uptake (VO_2_max·BM^-1^), were as follows: 59.75±2.05 kg, 166.63±1.14 cm and 44.8±1.1 mL·kg^-1^·min^-1^ in women, and 77.13±2.71 kg, 180.05±1.65 cm and 55.6±1.8 mL·kg^-1^·min^-1^ in men.

### Study design

Each participant underwent two laboratory exercise tests. 1) The incremental test (IT) was performed on a treadmill to determine VO_2_max. 2) The anaerobic exercise test (AnEx) involved a 20-second-long sprint on a bicycle ergometer to determine peak and mean power and changes in oxidative stress indicators in the blood. The participant’s diet was assessed for a week prior to AnEx. Women underwent the IT and AnEx between days 6 and 9 of the follicular phase during two subsequent menstrual cycles. Men underwent the IT and AnEx in two-week intervals. All exercise tests were conducted before noon in neutral temperature conditions (20–22°C). The participants were asked not to perform intense physical exercise for the seven days prior to each laboratory test and not to consume caffeine or alcoholic beverages within the 24 hours prior to each laboratory test.

### Anthropometric measurements

During the participants’ first visit to the laboratory, BH was measured using a Martin anthropometer (Poland) with accuracy to 1 mm and BM was measured using a Jawon IOI-353 Body Composition Analyzer (Korea).

### Incremental test

The IT was performed on an h/p/Cosmos Saturn COS 10198 treadmill (Germany) at an angle of 0°. The exercise began with a four-minute warm-up at 7.0 km·h^-1^ for men and 6.0 km·h^-1^ for women. After the warm-up, running speed continued to increase every two minutes by 1.2 km·h^-1^ for men and 1.0 km·h^-1^ for women until volitional exhaustion. Each participant was assumed to have reached their VO_2_max when the following criteria were met: a plateau in oxygen uptake was reached, the respiratory exchange ratio (RER) exceeded 1.15, and heart rate (HR) was close to maximal HR. VO_2_ and RER were measured using a Medikro 919 ergospirometer (Finland). VO_2_max values were expressed relative to BM (VO_2_max·BM^-1^). HR was registered during the test using an S-610i Polar Elektro pulsometer (Finland).

### Anaerobic exercise test

AnEx was performed on an 824E Monark bicycle ergometer (Sweden). The test was preceded by a four-minute warm-up at 60 revolutions per minute with a load of 90 W for men and 60 W for women. Two maximal accelerations, each lasting five seconds, were used in the second and fourth minutes of the warm-up. AnEx began after four minutes of rest, and involved the participant reaching the maximal revolution rate as quickly as possible and maintaining it for as long as possible. The load amounted to 7.5% of BM for men and 6.5% of BM for women. The test lasted 20 seconds [33,34]. This test was used to reduce the share of aerobic processes in covering the energy expenditure [35]. The participants remained in a sitting position (the height of the bicycle seat was adjusted individually) and were enthusiastically encouraged throughout the test. After the test, revolution rate decreased to 60 per minute. The participants maintained this rate for three minutes without any load.

The ergometer was connected to a computer and equipped with a magnetic timer that measured the duration of each revolution (with accuracy to 0.001 second). Peak power (PP) and mean power (MP) were calculated automatically based on these measurements with the Staniak JBA MCE software (Poland). Power was expressed in absolute values and relative to BM (PP·BM^-1^; MP·BM^-1^).

### Biochemical assays

#### Blood sample collection and pre-analysis procedure

Venous blood was collected six times: prior to the AnEx (Rest) and 3 minutes (Rec 3), 15 minutes (Rec 15), 30 minutes (Rec 30), 60 minutes (Rec 60) and 24 hours (Rec 24h) after AnEx (during recovery). On the first day a cannula was inserted into the venous vessels in the inside area of the elbow. The cannula was washed after insertion and before and after each blood collection with saline (1 ml of 0.9% NaCl) to prevent coagulation. The cannula was sealed with a catheter after collecting the blood for biochemical analyses. A total of seven milliliters of blood were collected each time. The first milliliter collected was always excluded from analysis. The remaining 6 ml were collected as three samples of 2 ml into tubes that contained: 1) blood coagulation activator, for measuring the concentration of uric acid (UA) in serum; 2) K_2_EDTA, for measuring the concentrations of reduced glutathione (GSH) and oxidized glutathione (GSSG) in whole blood, the concentrations of vitamins A (Vit A) and E (Vit E) in blood plasma, total oxidative status (TOS) of blood plasma, and total antioxidative capacity (TAC) of blood plasma; and 3) lithium heparin, for measuring the concentration of vitamin C (Vit C) in blood plasma. Immediately after the blood was collected, a pyridine derivative was added to GSSG measurement tubes as a thiol scavenger in order to prevent additional *in vitro* oxidation of GSH into GSSG. This derivative reacts quickly with GSH but does not interfere with glutathione reductase activity. Blood samples taken to obtain serum were centrifuged after being stored for 20 minutes at room temperature. Blood samples taken to obtain blood plasma were stored in the dark on ice and centrifuged as soon as possible. Centrifugation was performed for 15 minutes at 4°C and an RCF of 1.000×g in an MPW 351R centrifuge (Poland). Blood plasma, serum and full blood samples were stored prior to analysis at -70°C in a ULF 390 Arctiko freezer (Denmark).

Arterialized blood was collected from the fingertip twice: prior to (Rest) and three minutes after AnEx (Rec 3) into 300 μl tubes with K_2_EDTA and sodium fluoride as a glycolysis inhibitor for measuring lactate (Lac) concentration in blood plasma, and into 80 μl capillary tubes with lithium heparin as an anticoagulant for measuring hydrogen ions (H^+^) concentration in whole blood. The collected blood was centrifuged for three minutes at an RCF of 14.300×g in an MPW 55 centrifuge (Poland) in order to obtain blood plasma.

Blood samples were collected using BD equipment: cannulas, catheters, single-use syringes with saline, a set of vacuum tubes and microtubes (United States).

#### Biochemical analysis

*Lactate*. Lac concentration was measured through enzymatic colorimetry using a Randox L-Lactate assay (UK). Assay sensitivity amounted to 0.165 mmol·L^-1^. The assay was linear up to 19.7 mmol·L^-1^. Absorbance was measured at 550 nm using a Thermo Scientific Evolution 201 UV/VIS spectrophotometer (United States). Post-exercise increases in Lac concentration (ΔLac) were calculated.

*Hydrogen ions*. H^+^ concentration was measured immediately after collecting blood samples using a Siemens RapidLab 348 analyzer (Germany). Post-exercise increases in H^+^ concentration (ΔH^+^) were calculated.

*Total oxidative status and total antioxidative capacity*. TOS and TAC in blood plasma were measured five times (Rest, Rec 3, Rec 15, Rec 30 and Rec 60) using colorimetric method with the enzymatic PerOx Kit (KC5100) and ImAnOx Kit (KC 5200) assays developed by Immundiagnostik (Germany). TOS was determined based on the total content of lipid peroxides in a given sample. TAC of a given sample was determined by adding a known amount of H_2_O_2_, allowing the elimination reaction by antioxidants to occur for a particular time and then measuring the amount of remaining H_2_O_2_. The level of TAC was calculated based the difference between the added and remaining amounts of H_2_O_2_ following elimination. Measurements of both TOS and TAC involved the oxidation of tetramethylbenzidine (TMB) into a colored product that was subsequently measured using photometry. Absorbance was measured at 450 nm using a DRG MedTek E-Liza Mat 3000 microplate reader (USA). Test sensitivity amounted to 7 μmol·L^-1^ for TOS and 130 μmol·L^-1^ for TAC. The oxidative stress index (OSI) was calculated as the ratio of TOS to TAC (OSI = TOS/TAC).

*Antioxidant vitamins A*, *E*, *and C*. The concentrations of Vit A, Vit E, and Vit C in blood plasma were measured five times (Rest, Rec 3, Rec 15, Rec 30 and Rec 60) using an Immundiagnostik HPLC Kit (KC 1600) assay for vitamins A and E and an Immundiagnostik HPLC Kit (KC 2900) assay for vitamin C (Germany). Plasma samples were deproteinized and centrifuged for 10 minutes at an RCF of 10.000×g before measurements. Test sensitivity equaled 0.05 mg·L^-1^ for vitamin A, 1.00 mg·L^-1^ for vitamin E and 0.58 mg·L^-1^ for vitamin C.

*Uric acid*. UA concentration in serum was measured six times (Rest, Rec 3, Rec 15, Rec 30, Rec 60 and Rec 24h) using colorimetric method with the enzymatic Randox Uric Acid assay (UK). The assay was linear up to 1.19 mmol·L^-1^. Absorbance was measured at 520 nm using a Thermo Scientific Evolution 201 UV/Vis spectrophotometer (USA).

*Reduced and oxidized glutathione*. Samples were deproteinized using a frozen 5% solution of metaphosphoric acid (5% MPA). GSH and GSSG concentrations in the blood were measured six times (Rest, Rec 3, Rec 15, Rec 30, Rec 60 and Rec 24h) using an OBR, Inc. GSH/GSSG GT35 cuvette kit (United States) by enzymatic colorimetry assay according to the manufacturee’s instructions. The measurement involved the reduction of GSSG into GSH by glutathione reductase in the presence of NADPH, followed by the reaction of thiol groups with 5,5'-dithiobis-(2-nitrobenzoic acid) (DTNB). The rate of the reaction was proportional to GSH and GSSG concentrations. The colored product was then analyzed photometrically at 412 nm through a kinetics measurement using a Thermo Scientific Evolution 201 UV/VIS spectrophotometer (United States). The results were compared to the calibration curve for GSH and GSSG concentrations.

#### Diet analysis

During the seven days preceding the exercise test, participants applied a standardized diet in terms of the percentage of consumed protein (15%), fat (30%) and carbohydrates (55%) in meeting energy needs (2,700 kcal/day), and in terms of A, E and C antioxidant vitamin content dosages: 630μg/day, 10 mg/day and 75 mg/day. Each participant received menus prepared on the basis of the nutritional value of foods and dishes proposed by the Food and Nutrition Institute in Poland. The participants’ diet was assessed based on dietary diaries they were asked to keep and photographic records of food eaten [36] for the seven days prior to AnEx [37]. The assessment was conducted using the Dieta 5.0 (Food and Nutrition Institute, Poland) software (Table 1).

**Table 1:** Overview of participants’ daily diet.

Variable	Men (n = 10)	Women (n = 10)	Sex differences p—value
**Energy intake (kcal)**	2812.21±234.27	2097.16±123.17	0.015
**Protein (%)**	16.36±0.62	14.73±0.70	0.101
**CHO (%)**	38.98±0.70	32.78±1.59	0.002
**Fat (%)**	45.90±1.15	52.31±1.47	0.003
**Vit A (mg)**	1.07±0.15	0.82±0.13	0.240
**Vit E (mg)**	11.71±1.86	9.25±1.04	0.263
**Vit C (mg)**	82.77±12.09	136.33±20.03	0.034

Data are given as mean±standard error;

p < 0.05 statistically significant differences.

#### Statistical analysis

Data distribution was assessed with the Shapiro–Wilk test. The significance of differences by sex for one-time measurements was assessed with either the *t*-test for independent samples or the Mann–Whitney *U* test, depending on the distribution of variables. Sex differences in post-exercise changes of biochemical indicator concentrations were compared using multiple analysis of variance (MANOVA). If a main factor (sex, anaerobic exercise, or sex and anaerobic exercise) was found to be significant, the significance of differences between appropriate means was assessed using *post hoc* analysis (Tukey’s test and planned comparisons). For all variables, differences were assumed to be statistically significant at p <0.05. All data were presented as mean±standard error. The Statistica 10 (Stat-Soft, Inc., United States) software was used to perform calculations and draw charts.

## Results

**Fig 1:**
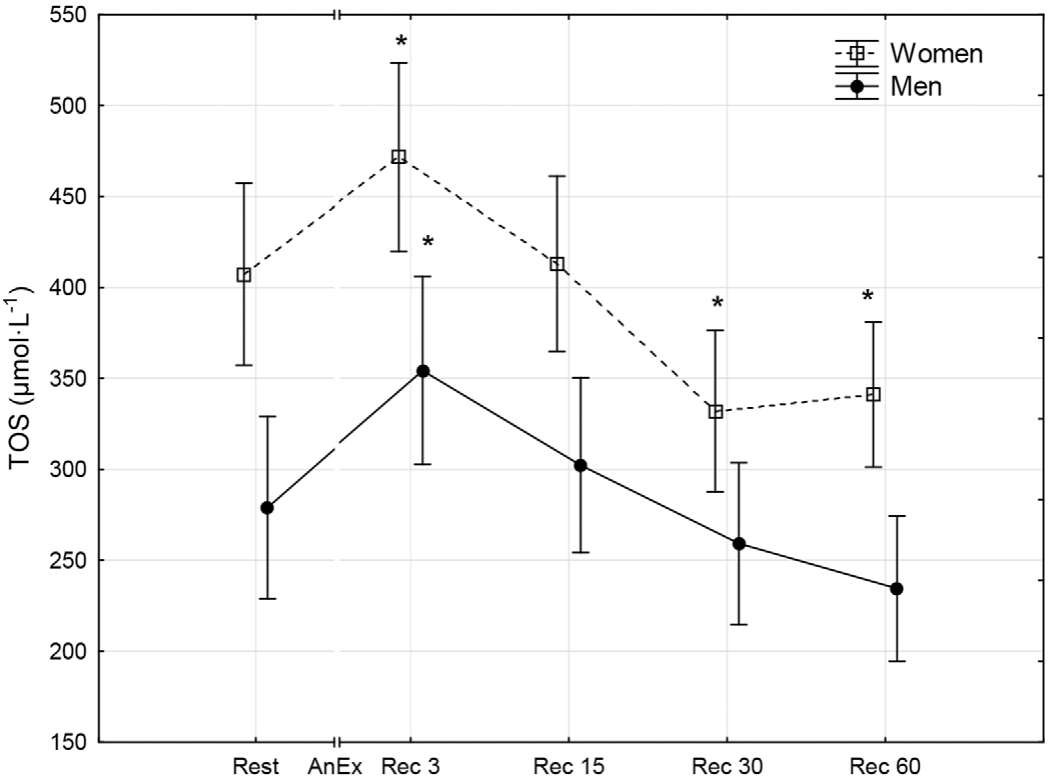
The level of total oxidative status (TOS) prior to (Rest) and 3 minutes (Rec 3), 15 minutes (Rec 15), 30 minutes (Rec 30) and 60 minutes (Rec 60) after anaerobic exercise (AnEx). Data are given as mean±standard error; *p < 0.05 statistically significant differences between results obtained after anaerobic exercise (AnEx) and initial results (Rest).

**Fig 2:**
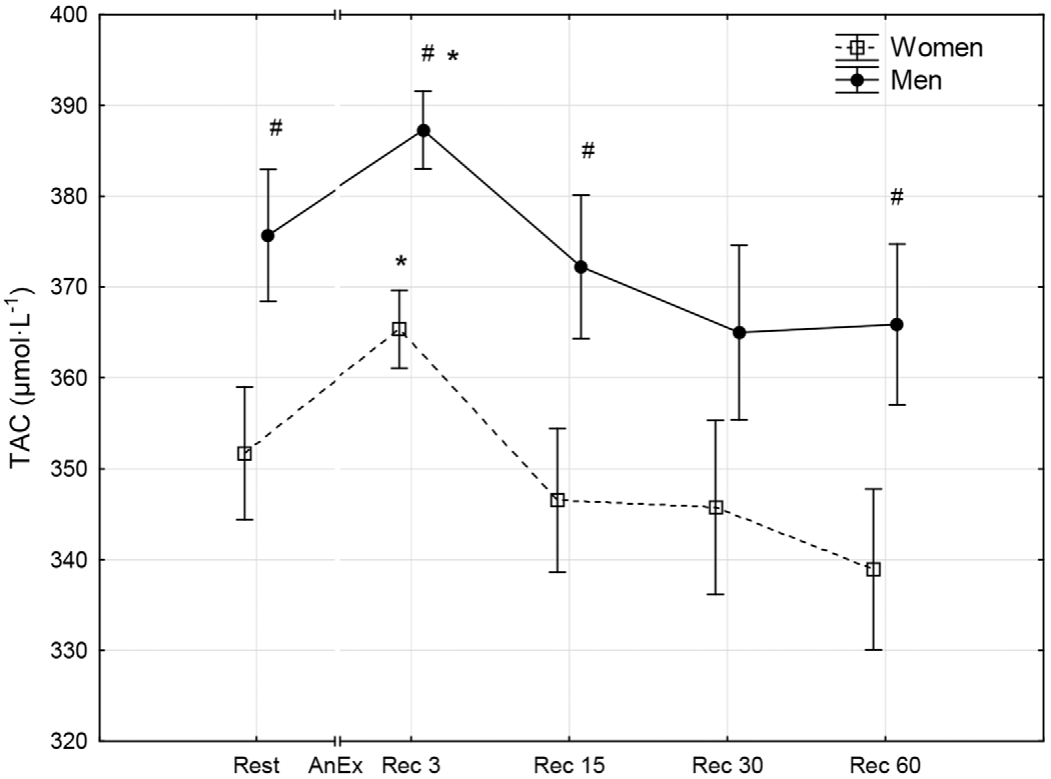
The level of total antioxidative capacity of blood plasma (TAC) prior to (Rest) and 3 minutes (Rec 3), 15 minutes (Rec 15), 30 minutes (Rec 30) and 60 minutes (Rec 60) after anaerobic exercise (AnEx). Data are given as mean±standard error; p < 0.05 statistically significant differences *between results obtained after anaerobic exercise (AnEx) and initial results (Rest); ^#^between results obtained by men and women.

**Fig 3:**
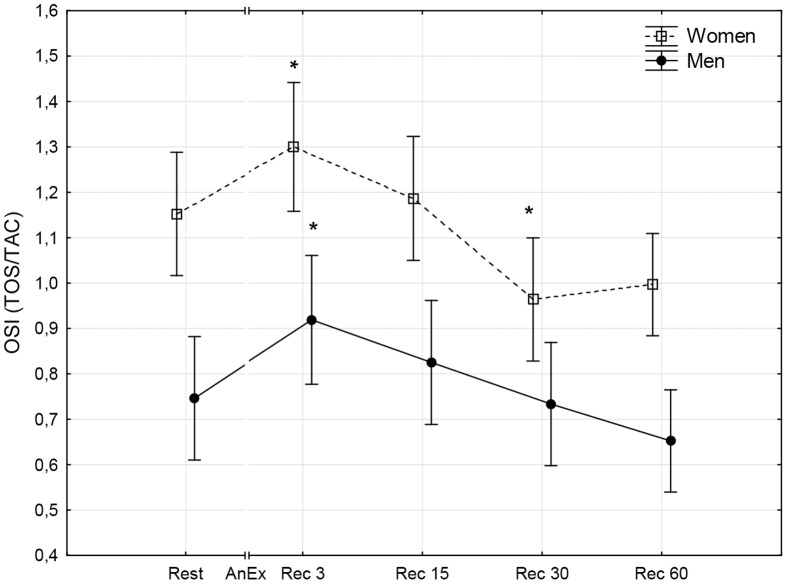
The level of the oxidative stress index (OSI) prior to (Rest) and 3 minutes (Rec 3), 15 minutes (Rec 15), 30 minutes (Rec 30) and 60 minutes (Rec 60) after anaerobic exercise (AnEx). Data are given as mean±standard error; *p < 0.05 statistically significant differences between results obtained after anaerobic exercise (AnEx) and initial results (Rest).

**Fig 4:**
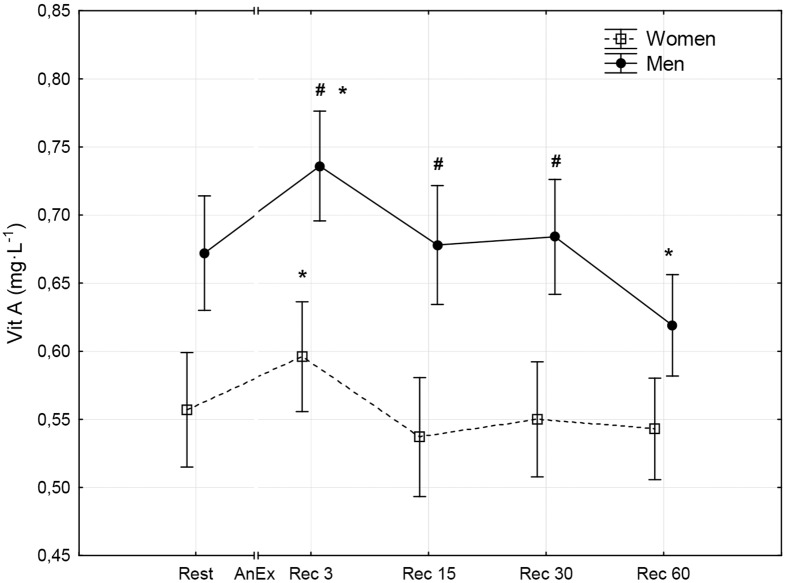
Concentration of vitamin A (Vit A) in blood plasma prior to (Rest) and 3 minutes (Rec 3), 15 minutes (Rec 15), 30 minutes (Rec 30) and 60 minutes (Rec 60) after anaerobic exercise (AnEx). Data are given as mean±standard error; p < 0.05 statistically significant differences *between results obtained after anaerobic exercise (AnEx) and initial results (Rest); ^#^between results obtained by men and women.

**Fig 5:**
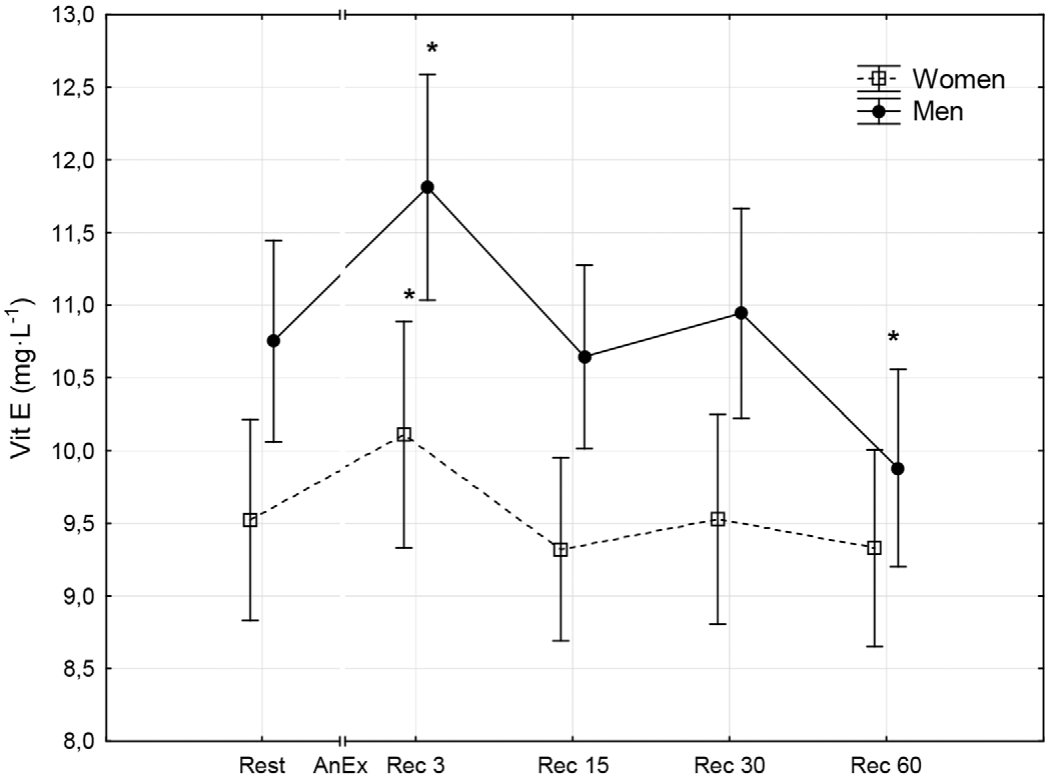
Concentration of vitamin E (Vit E) in blood plasma prior to (Rest) and 3 minutes (Rec 3), 15 minutes (Rec 15), 30 minutes (Rec 30) and 60 minutes (Rec 60) after anaerobic exercise (AnEx). Data are given as mean±standard error; *p < 0.05 statistically significant differences between results obtained after anaerobic exercise (AnEx) and initial results (Rest).

**Fig 6:**
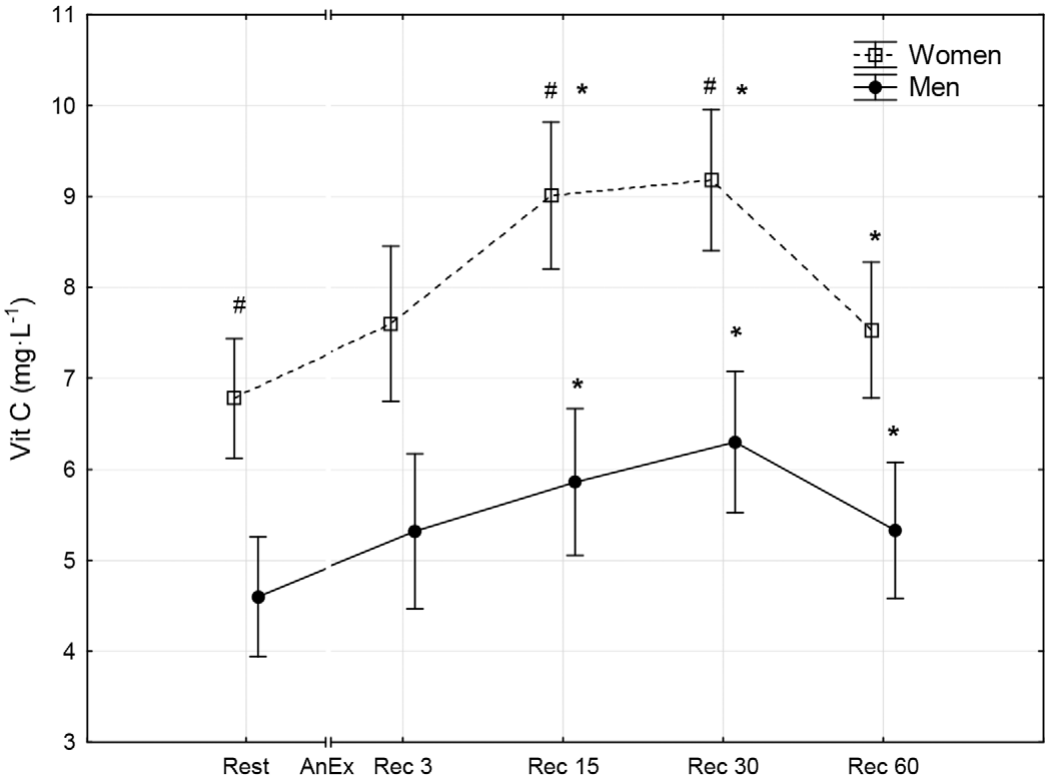
Concentration of vitamin C (Vit C) in blood plasma prior to (Rest) and 3 minutes (Rec 3), 15 minutes (Rec 15), 30 minutes (Rec 30) and 60 minutes (Rec 60) after anaerobic exercise (AnEx). Data are given as mean±standard error; p < 0.05 statistically significant differences *between results obtained after anaerobic exercise (AnEx) and initial results (Rest); ^#^between results obtained by men and women.

**Fig 7:**
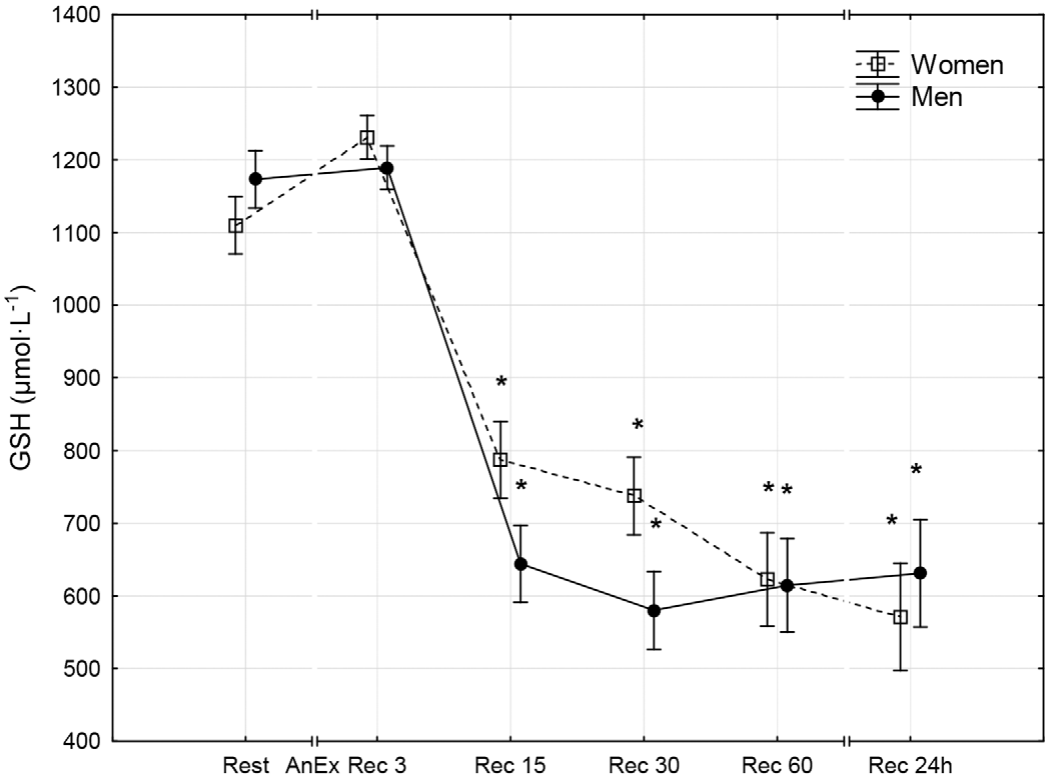
Reduced glutathione concentration (GSH) in the blood prior to (Rest) and 3 minutes (Rec 3), 15 minutes (Rec 15), 30 minutes (Rec 30) and 60 minutes (Rec 60) after anaerobic exercise (AnEx). Data are given as mean±standard error; *p < 0.05 statistically significant differences between results obtained after anaerobic exercise (AnEx) and initial results (Rest).

**Fig 8:**
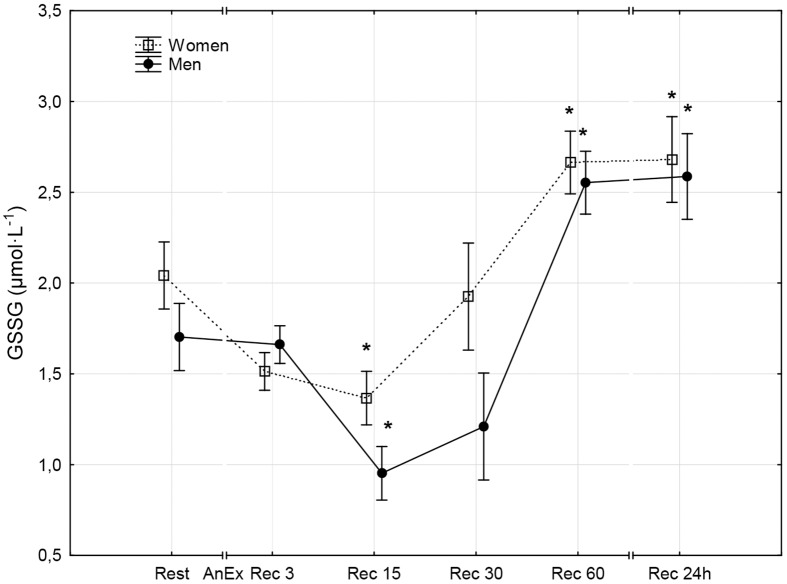
Oxidized glutathione concentration (GSSG) in the blood prior to (Rest) and 3 minutes (Rec 3), 15 minutes (Rec 15), 30 minutes (Rec 30) and 60 minutes (Rec 60) after anaerobic exercise (AnEx). Data are given as mean±standard error; *p < 0.05 statistically significant differences between results obtained after anaerobic exercise (AnEx) and initial results (Rest).

**Fig 9:**
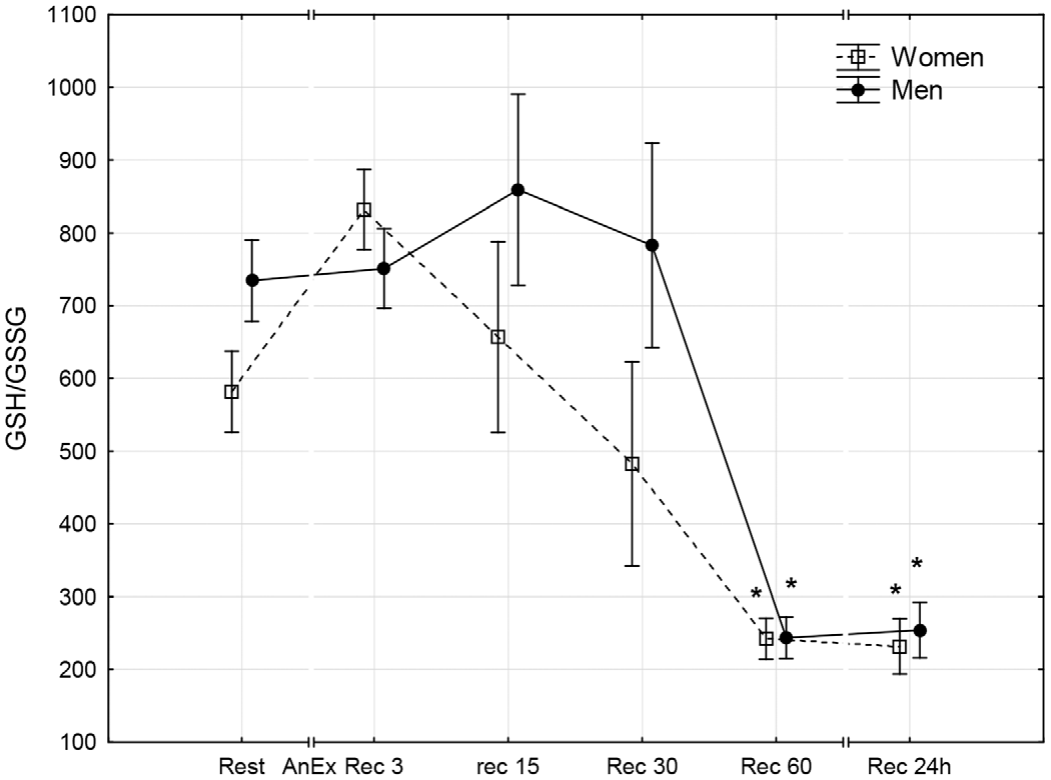
The reduced/oxidized glutathione ratio (GSH/GSSG) in the blood prior to (Rest) and 3 minutes (Rec 3), 15 minutes (Rec 15), 30 minutes (Rec 30) and 60 minutes (Rec 60) after anaerobic exercise (AnEx). Data are given as mean±standard error; *p < 0.05 statistically significant differences between results obtained after anaerobic exercise (AnEx) and initial results (Rest).

**Fig 10:**
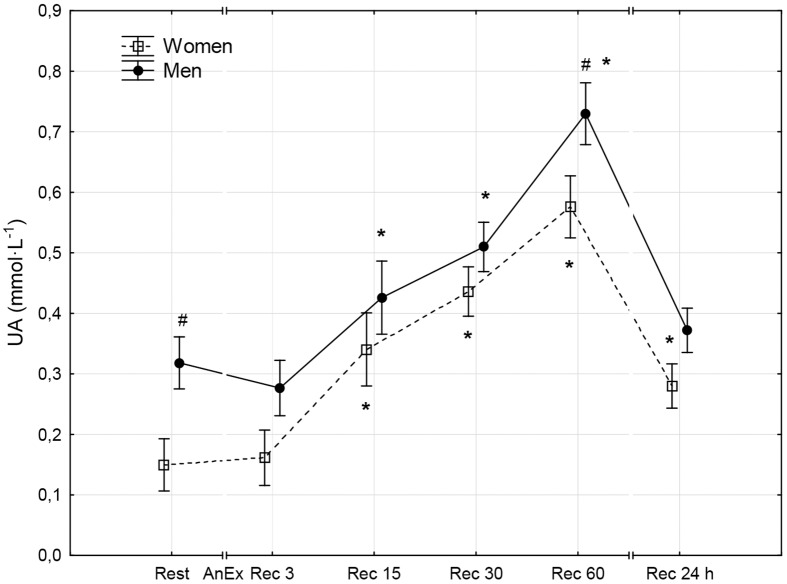
Uric acid concentration (UA) in the serum prior to (Rest) and 3 minutes (Rec 3), 15 minutes (Rec 15), 30 minutes (Rec 30) and 60 minutes (Rec 60) after anaerobic exercise (AnEx). Data are given as mean±standard error; p < 0.05 statistically significant differences *between results obtained after anaerobic exercise (AnEx) and initial results (Rest); ^#^between results obtained by men and women.

Men achieved significantly higher values of PP and MP (absolute and relative to BM) during AnEx than women (Table 2). The increase in H^+^ concentration in the blood and Lac concentration in blood plasma following AnEx was significant (p < 0.001) and did not differ significantly between sexes (ΔH^+^: 23.68±3.45 nmol·L^-1^ in men and 17.70±1.29 nmol·L^-1^ in women; ΔLac: 12.21±0.48 mmol·L^-1^ in men and 11.00±0.77 mmol·L^-1^ in women). Significant post-exercise changes in the concentrations of all oxidative stress indicators were found in men and women (Table 3; Figs 1–10). Changes in the concentrations of the analyzed oxidative stress indicators were similar in both sexes. No significant sex-anaerobic exercise interaction was found (Table 3). Both sexes showed the highest concentrations of TOS, TAC, Vit A and Vit E in blood plasma and OSI three minutes after AnEx (Rec 3). Vit C concentration in blood plasma and UA concentration in the serum increased continuously following AnEx and reached maximal values 30 and 60 minutes after AnEx, respectively, in both sexes. Beginning with the 15th minute after AnEx, both sexes showed a significant decrease (p < 0.001) in GSH concentration in whole blood relative to the initial value. The decrease lasted for 24 hours after AnEx. GSSG concentration in the blood significantly decreased 15 minutes after AnEx in both sexes. It subsequently began to increase, reaching a level significantly higher than the resting value, both after 60 minutes and 24 hours of recovery. The ratio of GSH/GSSG concentrations was significant lower than the resting value 60 minutes and 24 hours after AnEx. Vit A and TAC concentrations were significantly higher in men, while Vit C concentration was significantly higher in women.

**Table 2:** Absolute (W) and relative (W·kg^-1^) values of peak and mean anaerobic power (PP and MP) achieved during a 20-second anaerobic exercise (AnEx) by men and women.

Variable	Men (n = 10)	Women (n = 10)	Sex differences p—value
**PP (W)**	870.60±39.53	513.30±19.78	< 0.001
**PP (W·kg** ^**-1**^ **)**	11.28±0.26	8.59±0.14	< 0.001
**MP (W)**	724.90±29.55	438.20±18.56	< 0.001
**MP (W·kg** ^**-1**^ **)**	9.41±0.20	7.33±0.16	< 0.001

Data are given as mean±standard error;

p < 0.05 statistically significant differences.

**Table 3:** Effect of sex, exercise or the sex-anaerobic exercise interaction on biochemical indicators of oxidative stress.

	p value (MANOVA)		
Variable	Sex	Anaerobic Exercise	Sex—Anaerobic Exercise interaction
**TOS (μmol·L** ^**-1**^ **)**	0.109	<0.001	0.465
**TAC (μmol·L** ^**-1**^ **)**	0.004	0.007	0.981
**OSI**	0.067	<0.001	0.457
**Vit A (mg·L** ^**-1**^ **)**	0.044	<0.001	0.069
**Vit E (mg·L** ^**-1**^ **)**	0.210	<0.001	0.086
**Vit C (mg·L** ^**-1**^ **)**	0.026	<0.001	0.162
**UA (mmol·L** ^**-1**^ **)**	0.057	<0.001	0.403
**GSH (μmol·L** ^**-1**^ **)**	0.436	<0.001	0.073
**GSSG (μmol·L** ^**-1**^ **)**	0.062	<0.001	0.319
**GSH/GSSG**	0.120	<0.001	0.200

p < 0.05 statistically significant differences;

TOS—total oxidative status, TAC—total antioxidative capacity, OSI—oxidative stress index (TOS/TAC), Vit A—vitamin A, Vit E—vitamin E, Vit C—vitamin C, UA—uric acid, GSH—reduced glutathione, GSSG—oxidized glutathione.

## Discussion

The aim of this study was to compare disruptions in men and women to the prooxidant-antioxidant balance in the blood that take place within the first hour and the 24 hours after AnEx. The study assessed oxidative stress based on analysis of changes in TOS and TAC of blood plasma, changes in the concentrations of selected low molecular weight non-enzymatic antioxidants (GSH, UA, Vit A, Vit E and Vit C), and changes in GSSG concentration. The study also assessed disruptions to the prooxidant-antioxidant balance based on the GSH/GSSG concentration ratio and the OSI. Obtained results confirm one of the two proposed hypotheses. In both sexes, anaerobic exercise was found to cause disruptions to the prooxidant-antioxidant balance in the blood that lasted up to 24 hours after the exercise. However, contrary to the other proposed hypothesis, the disruptions increased to a similar extent in both sexes. Changes in the concentrations of all oxidative stress indicators following AnEx were significant in both sexes. Apart from GSH concentration and the GSH/GSSH concentration ratio, all changes took place progressively. The analyzed biochemical indicators of oxidative stress reached maximal values at different times after AnEx. OSI, TOS, TAC and Vit A and Vit E concentrations reached a maximal value after three minutes of recovery. Vit C concentration reached a maximal value after 30 minutes of recovery and UA concentration reached a maximal value after 60 minutes of recovery. At the same time, Vitamin C concentration in the blood was significantly higher in women than in men in all measurements. This, however, did not affect the antioxidant capacity of blood plasma, which was higher in men than in women. Consequently, the higher level of vitamin C observed in women could have resulted from a higher intake of the vitamin in their diet, rather than from sex differences related to the response to AnEx [38]. A decrease in GSH concentration in the blood was observed 15 minutes after AnEx. The decrease remained at a similarly low value for the 24 hours after AnEx. Surprisingly, GSSG concentration decreased 15 minutes after AnEx and only increased at a later stage of recovery, reaching a value significantly higher than the resting value after 60 minutes of recovery. GSSG concentration still showed a significant increase 24 hours after AnEx. A decrease in the GSH/GSSG concentration ratio, observed after one hour and as much as 24 hours after recovery, indicated increasing oxidative stress following AnEx. The highest concentration of thiobarbituric acid reactive substances (oxidative stress indicator) was observed after one hour after exercise at 90%VO_2_max, preceded by a 45-minute submaximal exercise (70–75%VO_2_max) and performed until volitional exhaustion, and the greatest changes in TAC, GSH and GSSG concentrations and the GSH/GSSG concentration ratio occurred two hours after anaerobic exercise [39]. Thus, the time after which the greatest changes in individual oxidative stress indicators occur depends on the duration and intensity of exercise. The results of this study differ from those obtained in an earlier study by Karabulut [40], who compared changes in oxidative stress indicators in men and women occurring directly after anaerobic exercise, in particular, a 20-meter run at maximal speed. Karabulut [40] found an increase in malondialdehyde as a product of oxidative damage to lipids and a decrease in GSH concentration only in men, and no significant changes in the analyzed indicators in women. A study by Ilhan et al. [41], the participants of which comprised 30 men and 30 women who performed the 30-second Wingate test, found that the initial levels of lipid peroxidation products were higher in men, GSH concentration was higher in women and anaerobic exercise did not significantly affect the levels of the analyzed indicators in either of the sexes. However, neither Karabulut [40] nor Ilhan et al. [41] assessed disruptions to the acid-base balance following anaerobic exercise. A positive correlation between the post-exercise increase in TOS and the post-exercise increase in Lac concentration in the blood was found following the IT until volitional exhaustion [13]. Therefore, the level of oxidative stress (similar in both sexes) following AnEx observed in this study may have been caused by a lack of sex differences in the increase in Lac and H^+^ concentration after the exercise.

A study by Cuevas et al. [20], in which men performed anaerobic exercise for 30 seconds, observed a significant decrease in GSH concentration and an increase in the GSSG/GSH ratio lasting up to 60 minutes after the exercise, which indicated the occurrence of oxidative stress. Groussard et al. [42] observed a systematic increase in UA concentration and an increase in Vit C concentration in the blood in men that lasted 40 minutes after exercise; these results are similar to those obtained in this study. A decrease in GSH concentration, an increase in GSSG, UA and Vit C concentrations and a decrease in the GSH/GSSG concentration ratio observed in this study confirm earlier results [20,42]. Furthermore, these changes indicate that oxidative stress can last as long as 24 hours after AnEx.

Antioxidant vitamins constitute even as much as 25% of total antioxidant capacity [25]. Thus, a decrease in TAC of blood plasma observed 20 minutes after the Wingate test [29] may indirectly indicate that antioxidant vitamins take part in neutralizing ROS produced during anaerobic exercise. This study showed that hydrophobic antioxidant vitamins are the primary compounds responsible for maintaining the prooxidant-antioxidant balance following AnEx. A decrease in vitamin concentration and a concurrent decrease in TOS and OSI were observed in both sexes after 15 minutes of recovery following an initial increase in TAC and an increase in Vit A and Vit E concentrations in blood plasma.

However, there are some limits in the current study. Firstly, sex differences in VO_2_max. Secondly, the lack of measurements of concentrations of some indicators 24 hours after anaerobic exercise (TOS, TAC, Vit A, Vit E and Vit C).

## Conclusions

In sum, the results obtained in this study lead to the conclusion that under similar disruptions to acid-base balance between sexes, anaerobic exercise causes the same changes in total antioxidative capacity, total oxidative status, the oxidative stress index and non-enzymatic antioxidants of low molecular weight in both sexes. Changes in GSH, GSSG and ratio of GSH/GSSG concentrations indicate that oxidative stress lasts at least 24 hours following anaerobic exercise. The interpretation of the level of disruptions to the prooxidant-antioxidant balance following AnEx should take into account the fact that post-exercise changes in oxidative stress indicators occur at different times.

## Abbreviations

AnEx: anaerobic exercise (20-second bicycle ergometer sprint)
GSH: reduced glutathione
GSH/GSSG: reduced/oxidized glutathione ratio
GSSG: oxidized glutathione
H^+^: hydrogen ions
IT: incremental test
Lac: lactate
MP: absolute value of mean power
MP·BM^-1^: relative to body mass value of mean power
OSI: oxidative stress index
PP: absolute value of peak power
PP·BM^-1^: relative to body mass value of peak power
Rec: after AnEx
Rest: prior to AnEx
RNS: reactive nitrogen species
ROS: reactive oxygen species
TAC: total antioxidative capacity
TOS: total oxidative status
UA: uric acid
Vit A: vitamin A
Vit C: vitamin C
Vit E: vitamin E

## References

[1] Vincent S, Berthon P, Zouhal H, Moussa E, Catheline M, Bentue-Ferrer D, et al Plasma glucose, insulin and catecholamine responses to a Wingate test in physically active women and men. Eur J Appl Physiol 2004; 91: 15–21. 14551777 10.1007/s00421-003-0957-5

[2] Janssen I, Heymsfield S, Wang Z, Ross R. Skeletal muscle mass and distribution in 468 men and women aged 18–88 yr. J Appl Physiol 2000; 89: 81–8. 10904038 10.1152/jappl.2000.89.1.81

[3] Esbjornsson-Liljedahl M, Bodin K, Jansson E. Smaller muscle ATP reduction in women than in men by repeated bouts of sprint exercise. J Appl Physiol 2002; 93: 1075–83. 12183505 10.1152/japplphysiol.00732.1999

[4] Green HJ, Fraser IG, Ranney DA. Male and female differences in enzyme activities of energy metabolism in vastus lateralis muscle. J Neurol Sci 1984; 65: 323–31. 6238135 10.1016/0022-510x(84)90095-9

[5] Simoneau JA, Bouchard C. Human variation in skeletal muscle fiber-type proportion and enzyme activities. Am J Physiol 1989; 257: E567–72. 2529775 10.1152/ajpendo.1989.257.4.E567

[6] Staron RS, Hagerman FC, Hikida RS, Murray TF, Hostler DP, Crill MT, et al Fiber type composition of the vastus lateralis muscle of young men and women. J Histochem Cytochem 2000; 48: 623–9. 10769046 10.1177/002215540004800506

[7] Toft I, Lindal S, Bonaa KH, Jenssen T. Quantitative measurment of muscle fiber composition in a normal population. Muscle Nerve 2003; 28: 101–8. 12811780 10.1002/mus.10373

[8] Esbjornsson M, Sylven C, Holm I, Jansson E. Fast twitch fibers may predict anaerobic performance in both females and males. Int J Sports Med 1993; 14: 257–63. 8365833 10.1055/s-2007-1021174

[9] Quindry J, Miller L, McGinnis G, Irwin M, Dumke C, Magal M, et al Muscle-fiber type and blood oxidative stress after eccentric exercise. Int J Sport Nutr Exerc Metabol 2011; 21: 462–70. 10.1123/ijsnem.21.6.462 22089306

[10] Durackova Z. Some current insights into oxidative stress. Physiol Res 2010; 59: 459–69. 19929132 10.33549/physiolres.931844

[11] Morales-Alamo D, Ponce-Gonzalez JG, Guadalupe-Grau A, Rodriguez-Garcia L, Santana A, Cusso R, et al Critical role for free radicals on sprint exercise-induced CaMKII and AMPKα phosphorylation in human skeletal muscle. J Appl Physiol 2013; 114: 566–77. 10.1152/japplphysiol.01246.2012 23288553

[12] Powers SK, Jackson MJ. Exercise-induced oxidative stress: cellular mechanisms and impact on muscle force production. Physiol Rev 2008; 88: 1243–76. 10.1152/physrev.00031.2007 18923182 PMC2909187

[13] Wiecek M, Maciejczyk M, Szymura J, Szygula Z. Changes in oxidative stress and acid-base balance in men and women following maximal-intensity physical exercise. Physiol Res 2015; 64: 93–102. 25194128 10.33549/physiolres.932744

[14] Esbjornsson-Liljedahl M, Sundberg CJ, Norman B, Jansson E. Metabolic response in type I and type II muscle fibers during a 30-s cycle sprint in men and women. J Appl Physiol 1999; 87: 1326–32. 10517759 10.1152/jappl.1999.87.4.1326

[15] Weber CL, Schneider DA. Maximal accumulated oxygen deficit expressed relative to the active muscle mass for cycling in untrained male and female subjects. Eur J Appl Physiol 2000; 82: 255–61. 10958366 10.1007/s004210000214

[16] Fisher-Wellman K, Bloomer RJ. Acute exercise and oxidative stress: a 30 year history. Dyn Med 2009; 8:1: 10.1186/1476-5918-8-1 19144121 PMC2642810

[17] Fuentes T, Guerra B, Ponce-González JG, Morales-Alamo D, Guadalupe-Grau A, Olmedillas H, et al Skeletal muscle signaling response to sprint exercise in men and women. Eur J Appl Physiol 2012; 112: 1917–27. 10.1007/s00421-011-2164-0 21928060

[18] Chen ZP, McConell GK, Michell BJ, Snow RJ, Canny BJ, Kemp BE. AMPK signaling in contracting human skeletal muscle: acetyl-CoA carboxylase and NO synthase phosphorylation. Am J Physiol Endocrinol Metab 2000; 279: E1202–6. 11052978 10.1152/ajpendo.2000.279.5.E1202

[19] Taito S, Sekikawa K, Oura K, Kamikawa N, Matsuki R, Kimura T, et al Plasma oxidative stress is induced by single-sprint anaerobic exercise in young cigarette smokers. Clin Physiol Funct Imaging 2013; 33: 241–4. 10.1111/cpf.12007 23522019

[20] Cuevas MJ, Almar M, García-Glez JC, García-López D, De Paz JA, Alvear-Órdenes I, et al Changes in oxidative stress markers and NF-κB activation induced by sprint exercise. Free Radic Res 2005; 39: 431–9. 16028368 10.1080/10715760500072149

[21] Berzosa C, Cebrian I, Fuentes-Broto L, Gomez-Trullen E, Piedrafita E, Martinez-Ballarin E, et al Acute exercise increases plasma total antioxidant status and antioxidant enzyme activities in untrained men. J Biomed Biotechnol 2011; 10.1155/2011/540458 PMC3062968 21436993

[22] Groussard C, Rannou-Bekono F, Machefer G, Chevanne M, Vincent S, Sergent O, et al Changes in blood lipid peroxidation markers and antioxidants after a single sprint anaerobic exercise. Eur J Appl Physiol 2003; 89: 14–20. 12627300 10.1007/s00421-002-0767-1

[23] Rutkowski M, Grzegorczyk K. Adverse effects of antioxidative vitamins. International Journal of Occupational Medicine and Environmental Health 2012; 25: 105–21. 10.2478/S13382-012-0022-x 22528540

[24] Sacheck JM, Blumberg JB. Role of Vitamin E and Oxidative Stress in Exercise. Nutrition 2001; 17: 809–14. 11684385 10.1016/s0899-9007(01)00639-6

[25] Wayner DD, Burton GW, Ingold KU, Barclay LR, Locke SJ. The relative contributions of vitamin E, urate, ascorbate and proteins to the total peroxyl radical-trapping antioxidant activity of human blood plasma. Biochim Biophys Acta 1987; 924: 408–19. 3593759 10.1016/0304-4165(87)90155-3

[26] Morales-Alamo D, Calbet JAL. Free radicals and sprint exercise in humans. Free Radic Res 2014; 48: 30–42. 10.3109/10715762.2013.825043 23879691

[27] Baker JS, Bailey DM, Hullin D, Young I, Davies B. Metabolic implications of resistive force selection for oxidative stress and markers of muscle damage during 30 s of high-intensity exercise. Eur J Appl Physiol 2004; 92: 321–7. 15098126 10.1007/s00421-004-1090-9

[28] Morales-Alamo D, Ponce-Gonzalez JG, Guadalupe-Grau A, Rodríguez-Garcia L, Santana A, Cusso RM, et al Increased oxidative stress and anaerobic energy release, but blunted Thr172-AMPK phosphorylation, in response to sprint exercise in severe acute hypoxia in humans. J Appl Physiol 2012; 113: 917–28. 10.1152/japplphysiol.00415.2012 22858621

[29] Podgorski T, Kowalczyk K. Antioxidant potential changes in tennis sportsmen organisms after Wingate Test. Polish J Sport Med 2006; 22: 215–20.

[30] Bloomer RJ, Smith WA. Oxidative stress in response to aerobic and anaerobic power testing: influence of exercise training and carnitine supplementation. Res Sports Med 2009; 17: 1–16. 10.1080/15438620802678289 19266389

[31] Kurkcu R, Cakmak A, Zeyrek D, Atas A, Karacabey K, Yamaner F. Evaluation of oxidative status in short-term exercises of adolescent athletes. Biol Sport 2010; 27: 177–80.

[32] Sarkin J, Campbell J, Gross L, Roby J, Bazzo S, Sallis J, et al Seven-day physical activity recall. Med Sci Sports Exerc 1997; 29: 89–103.

[33] Laurent CM Jr, Meyers MC, Robinson CA, Green JM. Cross-validation of the 20-versus 30-s Wingate anaerobic test. Eur J Appl Physiol 2007; 100: 645–51. 17429677 10.1007/s00421-007-0454-3

[34] Dybek T, Szygula R, Klimek A, Tubek S. Impact of 10 sessions of whole body cryostimulation on aerobic and anaerobic capacity and on selected blood count parameters. Biol Sport 2012; 29: 39–43.

[35] Inbar O, Bar-Or O, Skinner JS. Characteristics of the Wingate Anaerobic Test: Relative Aerobic and Anaerobic Contributions In: Inbar O, Bar-Or O, Skinner JS editors. The Wingate Anaerobic Test. Champaign: Human Kinetics, 1996: 35–7.

[36] Szponar L, Wolnicka K, Rychlik E, editors. Album of photographs of food products and dishes. Institute of Food and Nutrition, Warsaw, 2012.

[37] Daya NE, McKeown N, Wong MY, Welch A, Bingham S. Epidemiological assessment of diet: a comparison of a 7-day diary with a food frequency questionnaire using urinary markers of nitrogen, potassium and sodium. Int J Eidemiol 2001; 30: 309–17. 10.1093/ije/30.2.309 11369735

[38] Sakano N, Wang DH, Takahashi N, Wang B, Sauriasari R, Kanbara S, et al Oxidative stress biomarkers and lifestyles in Japanese healthy people. J Clin Biochem Nutr 2009; 44: 185–95. 10.3164/jcbn.08-252 19308273 PMC2654475

[39] Michailidis Y, Jamurtas AZ, Nikolaidis MG, Fatouros IG, Koutedakis Y, Papassotiriou I, et al Sampling time is crucial for measurement of aerobic exercise-induced oxidative stress. Med Sci Sports Exerc 2007; 39: 1107–13. 17596778 10.1249/01.mss.0b013e318053e7ba

[40] Karabulut AB. Effect of Exhaustive Exercise on Oxidative Stress and Adenosine Deaminase Activities in Women Compared to Men. Journal of US –China Medical Science 2011; 8: 150–5.

[41] Ilhan N, Kamanli A, Ozmerdivenli R, Ilhan N. Variable effects of exercise intensity on reduced glutathione, thiobarbituric acid reactive substance levels, and glucose concentration. Arch Med Res 2004; 35: 294–300. 15325503 10.1016/j.arcmed.2004.03.006

[42] Groussard C, Machefer G, Rannou F, Faure H, Zouhal H, Sergent O, et al Physical fitness and plasma non-enzymatic antioxidant status at rest and after a Wingate test. Can J Appl Physiol 2003; 28: 79–92. 12671197 10.1139/h03-007

